# Cutaneous Melioidosis and Necrotizing Fasciitis Caused by *Burkholderia pseudomallei*

**DOI:** 10.3201/eid0911.030370

**Published:** 2003-11

**Authors:** Yi-Shi Wang, Chin-Ho Wong, Asok Kurup

**Affiliations:** * Changi General Hospital, Singapore; † Singapore General Hospital, Singapore

## Abstract

In areas where melioidosis is endemic, stress on the healthcare system is substantial. Because clinical manifestations are protean, the illness is difficult to diagnose, and cutaneous *Burkholderia pseudomallei* infections can progress to necrotizing fasciitis. While it is an uncommon complication of cutaneous melioidosis, necrotizing fasciitis is potentially fatal and requires successful management, including early diagnosis, appropriate antibiotics selection, and operative débridement.

Melioidosis is a disease caused by a gram-negative bacterium, *Burkholderia pseudomallei*. Endemic to Southeast Asia, Taiwan, China, Central and South America, and northern Australia, sporadic infections occur throughout the world, usually in travelers to the disease-endemic areas (*1*–*3*). Humans are usually infected by soil contamination of puncture wounds. Respiratory tract infection is the most common sign of this disease (*3*). Cutaneous melioidosis with necrotizing fasciitis is rare: we found no reports of cutaneous melioidosis with necrotizing fasciitis and only one previous report of melioidosis with necrotizing fasciitis, which complicated postoperative drainage of a parotid abscess (*4*).

## The Study

A 61-year-old Chinese man with a history of psoriasis and alcoholic liver cirrhosis sought treatment for left ankle swelling, erythema, and tenderness. He worked as a dishwasher in a restaurant and could not recall any antecedent trauma to the affected limb. He was febrile on admission and was empirically treated with intravenous penicillin and cloxacillin for cellulitis. Despite antimicrobial drug therapy, his lower limb infection progressed with formation of cutaneous blisters. A decision for wound débridement was made. During surgery, grayish necrotic fascia, extensive subcutaneous tissue necrosis, loss of resistance of the normally adherent superficial fascia to blunt dissection, and foul-smelling “dishwater” pus were noted. A diagnosis of necrotizing fasciitis was made.

The tissue specimen was spread onto blood agar plate (trypticase soy agar supplemented with 5% sheep blood, BBL [Becton, Dickinson and Company, Franklin Lakes, NJ]) and Ashdown’s media, a selective media for *B. pseudomallei*. Characteristic colonial morphologic findings of *B. pseudomallei* were noted on blood agar and Ashdown’s media. The organism’s identity was confirmed with biochemical tests (positive oxidase reaction, simmons citrate) and microscopic morphologic findings. (The automated blood culture system used was the BACTEC Fluorescent Series blood culture system [Becton, Dickinson and Company].) Tissue (taken during surgery) and blood cultures grew *B. pseudomallei* susceptible to ceftazidime, amoxicillin/clavulanic acid, imipenem, chloramphenicol, and cotrimoxazole but resistant to ampicillin, amikacin, and gentamicin. Results of melioidosis serologic testing were also positive. The patient’s chest roentgenogram was normal. A computed tomography scan of his abdomen and pelvis showed splenic abscesses, but no prostatic abscess was noted. A digital rectal examination demonstrated an enlarged prostate that was nontender. The patient underwent two further operations for wound débridement and split thickness skin grafting. He was treated with ceftazidime and doxycycline; when his fever persisted, his treatment was converted to intravenous imipenem. He responded well and was discharged after he completed a 4-week course of imipenem. On discharge, no oral antimicrobial drugs were dispensed.

One month later, the man was readmitted with fever associated with scrotal swelling and pain. On examination, a firm, tender right prostatic nodule was noted. A transrectal ultrasound showed abscesses in both prostatic lobes. The aspirate from the prostatic abscesses and blood cultures grew *B. pseudomallei* with antimicrobial drug susceptibilities identical to the organism cultured from his initial admission. A 6-week course of intravenous ceftazidime was given. He also underwent transurethral resection of the prostate; the histologic results indicated benign prostatic hyperplasia with acute or chronic inflammation. On discharge, he was prescribed oral amoxicillin-clavulanate (for 4 weeks) and doxycycline (for 1 year).

Seventeen months after cessation of oral antibiotics, he reappeared with a left temporal scalp abscess and fever. No organism was isolated from the scalp pus culture, but the blood culture again grew *B. pseudomallei* with identical antimicrobial drug susceptibilities. He was given 4 weeks of ceftazidime followed by amoxicillin-clavulanate (for 4 weeks) and doxycycline (long-term). After 8 months on oral doxycycline, the patient was symptom-free.

## Conclusions

This patient history is representative of the protean clinical features of melioidosis. The disease has been called the great mimicker, with a wide range of clinical syndromes including pneumonia, visceral abscesses, soft tissue infections, septic arthritis, and septicemia (*1*,*3*,*5*). Lim et al. previously reported a case of melioidosis with a parotid abscess that was drained and subsequently progressed to necrotizing fasciitis (*4*). Although not previously reported, necrotizing fasciitis can be the initial sign of melioidosis. While cutaneous infections by *B. pseudomallei* are usually indolent soft tissue infections manifesting as cellulitis or abscesses (*1*–*3*,*6*), these soft tissue infections can progress to necrotizing fasciitis in melioidosis.

Melioidosis can remain latent for prolonged periods and reactivate with severe sepsis years after the initial infection (*1*,*7*). Melioidosis is characterized by relapses or recrudescence, especially in immunocompromised patients (*1*,*7*,*8*). For this patient, the first relapse occurred 1 month after the first hospital discharge. Another relapse occurred 17 months after cessation of oral maintenance antimicrobial drug therapy.

For patients with a known history of melioidosis, antimicrobial agents for *B. pseudomallei* should be used. B. pseudomallei are frequently intrinsically resistant to many antimicrobial agents, including aminoglycosides and first- or second-generation cephalosporins. Current recommended therapy of severe melioidosis includes intravenous ceftazidime or imipenem for 10 days to 4 weeks, followed by maintenance oral antimicrobial agents (amoxicillin-clavulanate or a combination of trimethoprim-sulfamethoxazole and doxycycline) for 10 to 18 weeks (*9*).

In the context of soft tissue infections, patients with a history of melioidosis developing cutaneous septic foci should be treated promptly and aggressively with antibiotics against *B. pseudomallei* in addition to the common organisms responsible for these infections such as *Staphylococci aureus* and *Streptococcal* species. In this setting, an intravenous regime consisting of crystalline penicillin and cloxacillin may not be the appropriate initial antimicrobial drug regime (*3*). Awareness that soft tissue infection can progress to necrotizing fasciitis, a life- and limb-threatening condition, is important, and a high index of suspicion should be maintained. When necrotizing fasciitis is suspected, magnetic resonance imaging of the affected area should be performed to ensure early diagnosis, and aggressive débridement of all nonviable tissue should follow for an improved outcome (*10*).

Melioidosis is a major problem and a rampant disease in rural parts of Central and South America, North Australia, and Southeast Asia. In northeast Thailand, it is responsible for 20% to 40% of deaths from community-acquired septicemia (*11*). Its true incidence is probably underestimated due to under detection and limited availability of culture facilities (*12*). Awareness of the spectrum of soft tissue infections caused by *B. pseudomallei,* including the distinct possibility of necrotizing fasciitis at the extreme of the spectrum, is important. The successful management of necrotizing fasciitis requires appropriate antimicrobial drug selection and aggressive operative débridement.
